# Initial stages of hand osteoarthritis do not affect the extrinsic muscles of the hand: a cross-sectional study

**DOI:** 10.1038/s41598-021-85054-3

**Published:** 2021-03-08

**Authors:** Natália Barbosa Tossini, André Luis Simões Zacharias, Luiza Souza Seraphim Abrantes, Paula Regina Mendes da Silva Serrão

**Affiliations:** Departament of Physical Therapy, Federal University of São Carlos, Highway Washington Luiz, km 235, São Carlos, SP CEP: 13565-905 Brazil

**Keywords:** Musculoskeletal system, Rheumatic diseases, Rehabilitation, Rheumatology, Signs and symptoms

## Abstract

The objective of this study was to verify whether women in the initial stages of hand osteoarthritis (HOA) already have impaired grip strength and flexor and extensor torque of the wrist compared to healthy women. It also aimed to correlate these variables with pain, stiffness, and function of the hand. Twenty-six women were divided into a control group [CG, n = 13; 56 (51–61) years old] and a hand osteoarthritis group [HOAG, n = 13; 58 (53–63) years old]. Grades II and III were included in the HOA group according to the criteria of Kellgren and Lawrence. All volunteers answered an initial assessment form, the Disabilities of the Arm, Shoulder, and Hand (DASH) questionnaire and the Australian/Canadian Hand Osteoarthritis Index (AUSCAN). The grip strength and isometric wrist flexor and extensor torque were evaluated by a hydraulic dynamometer. Comparisons between groups used Student’s *t* test for independent samples and the Mann–Whitney test. Spearman's correlation was used to correlate grip strength and flexor and extensor wrist torque to the degree of disease and DASH and AUSCAN scores. There were no differences between the groups in grip strength or flexor and extensor torque values. In terms of the DASH and the AUSCAN, the HOA group had higher scores, indicating worse hand function. A strong negative correlation was found between grip strength and the degree of HOA (r =  − 0.70, *p* = 0.008), and a moderate positive correlation was found between flexor torque and the degree of HOA (r = 0.53, *p* = 0.05). The pain (r =  − 0.61, *p* = 0.02) and function (r =  − 0.66, *p* = 0.01) sections of the AUSCAN correlated negatively with grip strength. Pain can be an important feature in the HOA rehabilitation process, as it can influence handgrip strength and function. It is important that rehabilitation is implemented as soon as possible to guarantee the maintenance of strength and function since with the severity of the disease, patients tend to have deficits in grip strength and function.

## Introduction

Osteoarthritis (OA) is a common and a complex chronic disease, characterized by pain and functional disability^1^. Hand osteoarthritis (HOA) is the most common form of OA. Its incidence is directly proportional to an increase in age and affects more women over 50 years old than men^2^. However, despite being the most prevalent form of OA, it has received little attention compared with hip and knee OA^3^.

HOA generally manifests itself as pain, stiffness, decreased range of motion, swelling, and joint damage that can lead to deformities and a decline in grip strength^4–6^. This clinical condition is accompanied by a functional compromise in these patients, who have difficulty performing activities related to personal care, household management, leisure activities and activities of daily living (ADL), such as eating, preparing meals, writing, buttoning and opening jars or bottles^7,8^. It has been demonstrated that activity limitations may deeply affect the quality of life of patients with hand OA^9,10^. Thus, the disease represents a major burden for both the individual and society because of its impact on the quality of life and the economic consequences linked to the loss of autonomy^11^.

Studies report that these functional changes are associated with deficits in pinch and grip strength, and the decline in grip strength can reach up to 60% compared with that of healthy subjects^4,5,12^. Several studies have analyzed the relationship between grip strength, disease severity and hand function^13–15^. These studies found a negative correlation between these variables^13–15^. This allows us to identify subjects with advanced degrees of HOA who have lower grip strength and worse hand function. However, it is not known whether this relationship occurs in the initial stages of the disease.

In addition, regarding hand function, it is important to underscore that wrist extensor muscles are an important component in performing ADLs, given that they stabilize the joint during functional activities involving pinching and/or grasping^16^. These muscles act in opposition to wrist flexors, preventing the wrist from flexing during grasping. If this occurs, the finger flexors enter active insufficiency, decreasing grip strength, which could compromise hand function^16^. However, information on wrist extension and flexion torque in subjects with HOA is scarce. Therefore, it is important to assess, in addition to grip strength, whether wrist flexor and extensor torque levels are compromised in patients with HOA, given the central role of extensors in joint stabilization and finger position during grip movements.

Thus, this study aimed to verify whether women in the initial stages of HOA already have impaired grip strength and flexor and extensor torque of the wrist compared to healthy women. It also aimed to correlate grip strength and flexor and extensor torque of the wrist with pain, stiffness, and function of the hand. We hypothesized that in the initial stages of HOA, grip strength and wrist extensor torque are reduced and correlate negatively with hand function; that is, the lower the grip strength, the greater the level of pain and the feeling of stiffness and the worse the self-report of hand function.

## Methods

### Participants

This is a cross-sectional study. Female participants over the age of 40 and with a medical diagnosis of HOA were included in the hand osteoarthritis group, according to the American College of Rheumatology (ACR) criteria^1^. The grading scales of HOA were confirmed by radiographic exam in the anteroposterior and oblique views (classified according to Kellgren and Lawrence’s criteria)^17^. The most affected interphalangeal joint was taken into account for the classification of the degree of disease, with women who presented grade II or III HOA included in this study. Healthy females of the same age were included in a control group that also underwent radiographic exams to confirm the absence of HOA.

The exclusion criteria for both groups were the presence of osteoarthritis at the base of the thumb, other rheumatic diseases, hand surgery, previous wrist or finger fracture and neurological and/or musculoskeletal disorders that could compromise upper limb function. The participants underwent the Mini Mental State Examination (MMSE) to ensure that none of them had cognitive impairment, and those who presented normal scores were included in the study^18^.

The data for this study were collected from January to October 2017 in the city of São Carlos, São Paulo state, SP and were approved by the Human Research Ethics Committee of the Federal University of São Carlos (Protocol No. 1.714.242). All research was performed in accordance with relevant regulations, and all participants were informed about the procedures performed and signed a Free and Informed Consent Form.

### Questionnaires

All the participants responded to the Disabilities of the Arm, Shoulder, and Hand (DASH) questionnaire used to assess upper arm symptoms and dysfunction in heterogeneous populations^19^. This questionnaire consists of 30 questions scored from 1 to 5, where 1 indicates no difficulty or symptoms and 5 indicates the inability to perform the task or very severe symptoms. The total score ranges from 0 to 100, where 0 indicates no impairment and 100 indicates complete disabilities^19^. This study used the DASH questionnaire version translated and validated for Portuguese^20^.

The participants also responded to the Australian/Canadian Hand Osteoarthritis Index (AUSCAN), which is a specific self-applicable questionnaire for HOA. It was translated, adapted and validated for Brazil^21^. The pain section was composed of 5 questions. The stiffness section contained 1 question, and the physical function section during common daily activities contained 9 questions. All questions refer to the 48 h prior to the application of the questionnaire. Each question has a score between 0 and 4, where 0 indicates no difficulty and 4 indicates extreme difficulty. The higher the score in the section, the greater the level of pain, feeling of stiffness and the difficulty in performing the activities described in the questionnaire^21^.

### Grip strength assessment

Grip strength was evaluated using a manual hydraulic dynamometer (JAMAR Hydraulic Hand Dynamometer-Model PC-5030J1, Fred Sammons, Inc*.,* Burr Ridge*,* IL*:* USA). The American Hand Therapist Society (ASHT) recommends that the assessment be performed with the subject sitting in a chair without arms and feet on the floor, shoulder adducted, elbow flexed at 90°, forearm in neutral pronation position, and extended wrist between 0° and 30°^22^.

Positions used for strapping dynamometers were position II for women and position III for men or a position above or below these if subjects’ hands were larger or smaller than average^22^. Before assessment, subjects handled the dynamometer to familiarize themselves with the instrument. Three 6-s repetitions were performed, with a 1-min rest period between them, and verbal encouragement was provided throughout the procedure. The average of the three repetitions, normalized by body weight, was used for statistical analysis.

### Isometric assessment of wrist flexor and extensor torque

Isometric assessments of wrist flexor and extensor torque were conducted using an isokinetic dynamometer (Biodex Multi-Joint System, Biodex Medical Incorporation, New York, NY, USA). Individuals sat in the chair of the device, stabilized by belts around the torso and a pelvic belt. The forearm was supported and secured in pronation, with the elbow flexed at 90° (0° = complete extension) and the shoulder in a neutral position. The rotation axis of the dynamometer was aligned with the wrist joint axis, located between the proximal row of the carpal bones and the distal extremity of the radius, which corresponds to the radiocarpal joint. The hand was positioned with the wrist in a neutral position and holding the device handle.

To familiarize individuals with the equipment, the subjects performed 3 submaximal and 2 maximum wrist flexion and extension repetitions. Next, for the assessment itself, 5 maximum flexion and 5 maximum extension repetitions were performed, with a 2-min interval between each repetition. Two positions were considered to assess extensor torque, the first with the wrist in a neutral position and the second with the wrist extended at 15°, given that this is the functional position of the joint^12^. Verbal encouragement was provided during the test to stimulate the volunteers to produce maximum torque. The average isometric wrist flexor and extensor peak torque were used for data analysis.

### Statistical analysis

Statistical analysis was conducted using the Statistical Package for the Social Sciences software, version 19.0 (SPSS Inc, Chicago, IL, USA). Categorical data are presented as percentages. The normality of the data was verified through the Shapiro–Wilk test, and homoscedasticity was verified by the Levene test. When the results showed a normal distribution, groups were compared using Student’s *t* test for independent samples; on the other hand, when the results did not show such a distribution, the Mann–Whitney test was used for comparison. The Mann–Whitney test was used for grip strength, flexor and extensor torque and the AUSCAN and DASH questionnaires. For the characteristics and values of the wrist range of motion, Student’s *t* test was used.

Spearman's correlation was used to relate grip strength, flexor and extensor wrist torque to the degree of disease, DASH scores and sections of AUSCAN questionnaires. The r values were interpreted as follows: 0.00–0.19 = none-mild; 0.20–0.39 = low; 0.40–0.69 = moderate; 0.70–0.89 = strong and 0.9–1.00 = very strong^23^.

A 5% significance level was adopted for all analyses (α ≤ 0.05).

## Results

Eighteen women with HOA were initially evaluated; however, three of them were excluded due to diagnosis with another rheumatologic disease, and two were excluded due to the presence of osteoarthritis at the base of the thumb. Therefore, thirteen women with disease formed the HOA group (n = 13; 58.31 years). Thirteen healthy women formed the control group (n = 13; 56.31 years). All volunteers were right-handed. Table 1 shows the clinical characteristics and the ranges of motion (ROMs) of the wrist from both groups (values presented as the mean (ICC)). Notably, the HOA group had less ROM in the wrist extension than the control group. Of the thirteen patients evaluated in the HOA group, seven (53.84%) presented Heberden’s nodes, and six (46.15%) presented Heberden’s and Bouchard’s nodes. Seven (53.84%) patients were classified as having grade II disease, and six (46.15%) were classified as having grade III.

**Table 1:** Characteristics and values of the wrist range of motion in both groups.

*Characteristics*	*HOAG (n* = *13)*	*CG (n* = *13)*	*p*
Age (years)	58.31 (53–63)	56.31 (51–61)	0.53
Height (cm)	158 (155–160)	162 (158–165)	0.08
Weight (kg)	66.08 (58–73)	71.46 (64–78)	0.28
BMI (kg/cm^2^)	26.69 (23–29)	27.23 (24–29)	0.76
Flexion (°)	71 (64–78)	78 (73–83)	0.07
Extension (°)	59 (54–63)	68 (62–73)	0.01*
Ulnar deviation (°)	24 (20–29)	26 (21–32)	0.47
Radial deviation (°)	36 (30–43)	41 (35–46)	0.29

*Significant difference.

Upon analyzing the grip strength and the flexor and extensor torque values, we observed no difference between the groups (Table 2, values presented as the mean (ICC)). However, in relation to the DASH and all the sections of the AUSCAN questionnaires, the HOA group had higher scores, indicating more pain, more stiffness, and greater dysfunction than the control group.

**Table 2:** Force values and questionnaires.

	*HOAG (n* = *13)*	*CG (n* = *13)*	*p*
Grip strength (kgf)	33.05 (28–38)	32.09 (27–37)	0.44
Wrist flexor torque (Nm)	7.71 (6–9)	7.93 (5–10)	0.68
Wrist extensor torque 0° (Nm)	2.39 (1–3)	2.22 (1–2)	0.57
Wrist extensor torque 15° (Nm)	2.40 (1–3)	2.07 (1–2)	0.61
DASH	28.60 (17–40)	9.97 (− 4–24)	0.001*
AUSCAN pain section	9.38 (7–11)	0.08 (− 0.10–0.27)	≤ 0.001*
AUSCAN stiffness section	1.92 (1–2)	0 (0–0)	≤ 0.001*
AUSCAN function section	14.85 (9–20)	1.58 (0.39–2)	≤ 0.001*
AUSCAN total	26.15 (18–34)	1.67 (0.30–3)	≤ 0.001*

*Significant difference.

A strong negative correlation was found between grip strength and the degree of HOA (r =  − 0.70, *p* = 0.008), indicating an inverse relationship between these variables. The higher the degree of HOA was, the lower the patient's grip strength. A significant moderate positive correlation was also found between flexor torque and the degree of HOA (r = 0.53, *p* = 0.05), indicating a direct relationship between these variables; that is, the higher the degree of HOA was, the greater the flexor torque of the wrist.

The pain (r =  − 0.61, *p* = 0.02) and function (r =  − 0.66, *p* = 0.01) sections of the AUSCAN also correlated negatively with grip strength. These results indicated that the lower grip strength was, the greater the pain and physical dysfunction, as shown in Table 3.

**Table 3:** Correlations between grip strength and torque and variables in HOAG.

Variables	Grip strength	Flexor torque	Extensor torque	Extensor torque at 15°
Grade HOA	r = − 0.70 *p* = 0.008*	r = 0.53 *p* = 0.05*	r = − 0.12 *p* = 0.68	r = − 0.38 *p* = 0.19
DASH	r = − 0.44 *p* = 0.13	r = − 0.11 *p* = 0.72	r = 0.28 *p* = 0.33	r = 0.25 *p* = 0.40
AUSCAN pain section	r = − 0.62 *p* = 0.02*	r = 0.03 *p* = 0.90	r = − 0.05 *p* = 0.87	r = − 0.02 *p* = 0.94
AUSCAN stiffness section	r = − 0.53 *p* = 0.06	r = 0.15 *p* = 0.60	r = 0.48 *p* = 0.09	r = 0.36 *p* = 0.22
AUSCAN function section	r = − 0.66 *p* = 0.01*	r = 0.16 *p* = 0.59	r = 0.22 *p* = 0.43	r = 0.13 *p* = 0.67
AUSCAN total	r = − 0.70 *p* = 0.007*	r = 0.14 *p* = 0.63	r = 0.17 *p* = 0.57	r = 0.09 *p* = 0.75

*Significant difference.

## Discussion

The results of this study show that, despite not showing a deficit in grip strength in the intergroup comparison, we found that women with HOA have higher levels of pain, greater morning stiffness and more difficulty performing activities of daily living than healthy women. Furthermore, the results show that there is an indirect correlation between disease severity and grip strength, indicating that with HOA progression to more advanced stages, grip strength tends to decrease, and a direct correlation between disease severity and wrist flexor torque indicates that disease progression can generate adaptations in these muscles, such as the production of greater flexor torque to perform activities of daily living. The results also demonstrate that there was an indirect correlation between the AUSCAN questionnaire and grip strength, demonstrating that decreased grip strength impairs function and can increase pain levels.

### Grip strength and wrist muscle torque

The present study found that women with HOA in the initial degrees do not show reductions in grip strength or wrist extensor and flexor torque values compared to healthy women. Several studies have shown that osteoarthritis affects the function of the hand, especially in advanced degrees of the disease (grades 3 and 4)^12–15^. One of the variables that can influence hand function is grip strength. Grip strength is an important index of the functional integrity of the hand and a good indicator of the patient's overall muscle strength, and its maintenance is essential for the execution of manual tasks of daily life^24,25^. Our study found that there was no difference in grip strength in women with and without HOA, indicating that in the initial stages of the disease, there is still no impairment.

In addition, this study also found no difference between flexor and extensor torque in women with and without HOA, suggesting that in the initial stages, there is also no compromise of the muscle strength of this joint. However, the lack of data in the literature on this subject does not allow a comparison of data.

These results suggest that HOA in the initial degrees may not affect muscle components, be these intrinsic or extrinsic muscles of the hand.

### Functional questionnaires

Despite the absence of changes in muscle strength in these women with HOA, when assessing the patient's perception of her own pain and function, significant differences were found when compared to healthy women. Thus, even though there is no objective impairment of strength, these women already present subjective changes in pain and function, which can negatively influence quality of life. Several studies have also found worse self-reported function in patients with HOA. Buckhave and Huniche (2013) had already reported that people with HOA experienced problems of activity and social participation, mainly in self-care, work, and leisure activities that involve the grasping of objects^26^. According the answers in the function section of the AUSCAN questionnaire in our study, the activity of twisting clothes was identified with a higher difficulty score to be performed, followed by opening a new pot and lifting large/heavy objects. We suggest that what may influence these women to report difficulty in performing manual activities is pain.

It is worth highlighting the importance of these results for clinical practice, as they demonstrate that the HOA rehabilitation process goes beyond grip or pinch training. We emphasize the importance of paying attention to individual needs and focusing, in addition to the manual function, on the occupational performance of patients and strategies for coping with the disease.

### Correlations between disease severity, grip strength, pain and function

Several studies have also found a negative correlation between the degree of osteoarthritis and grip strength^12–15^. Bagis et al. (2003) found that the more radiographic changes and involvement of more than one hand joint, the lower the patient's grip strength^27^. Dominick et al. (2005) observed that a more severe classification, according to the KL criteria, was associated with lower grip and clamp strength, even in patients with self-reported pain control^13^. Our results indicate that this correlation between the severity of disease and grip force already exists in the initial degrees, indicating that with disease progression, this grip force tends to decrease, further damaging the function. It is worth noting that one limitation of the study was that we did not classify, according to Kellgren-Lawrence's criteria, all the affected joints of the hand.

The results also showed a negative correlation between pain and self-reported function and grip strength of women with HOA. Other studies in the literature also found this relationship^4,5^; however, the functional deficit reported by these studies was usually observed in patients with advanced degrees of the disease, suggesting that the severity of HOA also contributes to variations in function. However, in the present study, the variables disease severity and function were not correlated. Thus, we suggest that the relationship between grip force and function may also be influenced by the patient's level of pain. This is because our study found a negative correlation between grip force and pain, i.e., self-reported pain by the patient may generate deficits in grip force and consequently alter hand function. Jones et al. (2001)^12^ and Bagis et al. (2003)^27^ have already suggested that much of the effect of HOA on grip function and strength is mediated by pain.

These results are important because they reinforce that the rehabilitation process of patients with HOA needs to be broad and multidimensional. Despite the absence of grip strength deficits and wrist torque, women with HOA present functional deficits and pain. In the initial degrees of the disease, it is already possible to observe that the three variables (grip force, function and pain) correlate and influence each other. Thus, it is important that rehabilitation be implemented as soon as possible to guarantee the maintenance of strength and function since with the severity of the disease, patients tend to have a deficit in these variables.

Furthermore, regarding the DASH questionnaire, which assesses heterogeneous populations with upper limb dysfunction, no relationship was found. This suggests that both patient rehabilitation and evaluation must be based on evidence. In other words, it is important to use specific and appropriate tools for populations of patients with hand osteoarthritis.

Finally, we also found a positive relationship between the degree of HOA and the flexor torque of the wrist. There are no studies in the consulted literature that have evaluated this relationship for this population. Therefore, we suggest that this relationship may be due to muscle adaptation. Since with the progression of the disease, the tendency is a deficit in handgrip strength, these patients can try to compensate for this deficit by increasing the muscle torque of the wrist flexors to maintain efficiency in the execution of tasks. However, further studies are needed to investigate the effects of HOA on the wrist joint and their relationship to the disease severity.

### Points of weakness and strength

Additionally, the number of participants can be a limitation of this study. Despite the intense recruitment, it was difficult to find subjects to compose the control group, due to the need of absence of hand pain in this age range. Therefore, our sample was limited to 13 participants in each group.

Although we have some limitations, one of the strong points was the evaluation of the wrist flexor and extensor torques in people with HOA. Some articles have explored this variable in other health conditions, such as epicondylitis or neuromuscular diseases (i.e. limb-girdle muscular dystrophy). However, so far, the authors have not found any study that assessed wrist flexor and extensor torque in patients with HOA. Despite the absence of statistical difference in our study, new paths can be explored, as function and strength of the wrist influence the grip strength, which also can influence the function of the hand. Thus, we leave as a suggestion for future studies the assessment of the wrist flexor and extensor torque in a larger sample, to identify if there is influence of this variable on the function of the hand in patients with HOA.

Another strong point of the study is the inclusion of patients with HOA in initial degrees of the disease (degrees II and III). Most studies compare the clinical condition of patients between degrees and have observed that grade IV is worse than grade II. Our study compared strength and function of the hand between patients with HOA and controls without HOA, which allows us to state that there are significant differences in pain, function, and stiffness of the hand in the population with this disease. Also, the sample with only patients in initial stages of the disease allows us to recommend that early diagnosis and rehabilitation are important for this population because it is known that with the progression of the disease the clinical condition tends to worsen.

## Conclusion

We conclude that women with mild degrees of HOA present pain and functional deficits that are related to grip strength. Moreover, the severity of the disease influences the reduction in grip strength, which would consequently further affect pain and function.

## Data Availability

The datasets generated during and/or analyzed during the current study are available from the corresponding author on reasonable request.
